# Validity and Reproducibility of a Food Frequency Questionnaire as a Measure of Recent Dietary Intake in Young Adults

**DOI:** 10.1371/journal.pone.0075156

**Published:** 2013-09-18

**Authors:** Lana Hebden, Engracia Kostan, Fiona O’Leary, Allison Hodge, Margaret Allman-Farinelli

**Affiliations:** 1 The University of Sydney, School of Molecular Bioscience, Sydney, New South Wales, Australia; 2 The Cancer Council Victoria, Cancer Epidemiology Centre, Victoria, Australia; The Australian National University, Australia

## Abstract

This research assessed the relative validity and reproducibility of the Dietary Questionnaire for Epidemiological Studies (DQESV2) over one month in young adults, given the lack of concise and convenient instruments for assessing recent dietary intake in this population. Participants were recruited from a large Australian university (N = 102; 35% male; age 18–34 years; body mass index 16–37 kg/m^2^). Five one-day weighed food records (WFR) were administered over one month followed by the DQESV2. Estimates for nutrients (energy, protein, total fat, saturated fat, carbohydrate, sugars, dietary fibre, and alcohol) and fruit and vegetable servings were compared between methods using correlation coefficients, 95% limits of agreement, and quintile classifications. One week later, a second DQESV2 was completed by n = 77 of the participants to assess reproducibility using intra-class correlations (ICC) and weighted kappa. Comparing methods, all nutrients and fruit and vegetable servings showed significant positive correlations (*P*<0.05) except protein intake in males; over 60% of participants were within one quintile classification except total fat and dietary fibre intakes in males (55% and 56%, respectively); and differences in nutrient and food intakes between methods were all within +/−20% of the mean WFR values except alcohol intake in females. Between first and second administrations of the DQESV2 all ICC coefficients were positive (*P*<0.01) and weighted kappa coefficients ranged from 0.54 for fruit servings (including fruit juice) in males to 0.91 for protein intake in females. Over a one month period, the DQESV2 demonstrated good reproducibility for the studied nutrients and for fruit and vegetable servings and provided a valid measure of the studied nutrients, except alcohol in females, and of fruit servings (including fruit juice) in both genders, at the group level in this young adult population.

## Introduction

The food frequency questionnaire (FFQ) is a method of dietary assessment that measures an individual’s usual consumption of food items or food groups within a specified time period [1]. They are commonly used to measure dietary intake in large populations for epidemiological studies, as they place less burden on the respondent and are usually less expensive and more easily administered than other methods of dietary assessment [1], [2]. Selection of one of the numerous FFQs available internationally should be based on whether the questionnaire contains foods common to the study population demographic and food supply of the region. In Australia, there are a limited number of FFQs that are in common use, one being the Cancer Council Victoria Dietary Questionnaire for Epidemiological Studies (DQESV2) [3].

In the 1980’s the Cancer Council Victoria developed a 121 item FFQ to measure dietary intake in men and women aged 40 to 69 years, participating in the Melbourne Collaborative Cohort study [4]. The original FFQ was developed based on the reported food intakes of 810 healthy volunteers born in Australia, Greece and Italy in two four day weighed food records [4]. Since then, the FFQ has been modified to the Dietary Questionnaire for Epidemiological Studies (DQESV2), which has 74 food items and additional questions to improve the quantification of intake. The validity of the DQESV2 as a measure of nutrient intake has been evaluated over the preceding 12 months in women of mean (SD) age 33 (10) years [5], the preceding one month in adults aged 58 (9) years [6], and compared with biomarkers of dietary n-3 and n-6 fatty acids in adults aged 40 (6) years [7]. However, validity of the DQESV2 has yet to be evaluated in younger Australian men and women (ages 18 to 34 years), a population for which there is a lack of easily administered questionnaires that may be used to accurately assess dietary intake.

Compared with older adults, young adults consume the lowest amount of fruit and vegetables [8], and obtain a higher proportion of their energy from snack foods, confectionery and sugar-sweetened beverages [9]. Of particular concern are the dietary behaviours associated with weight gain during young adulthood (ages 18 to 34 years), given the high risk for overweight during this life stage [10]. These include inadequate fruit and vegetable intake and an excessive intake of high fat takeaways (fast-foods) and sugar-sweetened beverages [11]–[13]. Hence, nutrients that are associated with intakes of these foods such as total energy, saturated fats, added sugars, dietary fibre and alcohol, as well as fruit and vegetables as a food group, were of primary interest for evaluation in this study. As dietary intakes and food habits will differ by age, coupled with DQESV2 being designed for an older population, it was deemed appropriate to examine the relative validity and reproducibility of the Cancer Council Victoria DQESV2 as a measure of intake of the selected nutrients and foods in young adults.

## Methods

### Participants

Participants were recruited via student newsletters and advertisements posted around the main university campus during August, 2011. Prospective participants were screened for the following exclusion criteria: weight reducing or medical diet; pregnant; aged <18 or >34 years. Body weight was measured to nearest 0.1 kg using digital scales in light clothing, without shoes, having removed accessories e.g. watches, wallets. Standing height was measured to nearest 0.1 cm using a stadiometer, standing with a straight spine, gaze forward. All participants were reimbursed for their participation. This study was conducted according to the guidelines laid down in the Declaration of Helsinki and all procedures involving human subjects/patients were approved by the University of Sydney Human Research Ethics Committee (approval no. 13746). Written informed consent was obtained from all participants.

### Study Procedure

#### Weighed Food Records (WFR)

The WFR was selected as the reference method to validate the DQESV2 [1], [14]. Subjects completed five one-day WFR, including three weekdays and two weekend days. This protocol resulted in a total of 510 one-day WFRs for the sample, with representation from each day of the week (Monday 4%, Tuesday 6%, Wednesday 16%, Thursday 20%, Friday 20%, Saturday 20% and Sunday 14%); that is 34% of records were from weekends and 66% from weekdays. Each one-day WFR was completed five days apart over a one month period, i.e. after the first one-day WFR was completed the second was completed on the sixth day after the first. Five days of WFR were selected to provide a measure of usual intake, without being overly burdensome on participants. Given that our interest was in overall fruit and vegetable intake rather than specific varieties, and in macronutrients, five days of WFR is considered sufficient [15]. A blank food and beverage record booklet, scales, and household measuring cups and spoons were provided to assist in measuring intake. Detailed instructions were provided on weighing foods and beverages consumed at home and estimating intake using household measures when dining out. When subjects ate out, household measures were converted to gram weights in accordance with the Australian food measures database [16]. Participants were advised to record detailed descriptions of foods and beverages (types, amounts, brands, time and location, meal or snack occasion, cooking methods and recipes used). Mobile phone text messages were sent to participants on the day prior to each day of recording as a reminder. A dietitian checked completed records for errors and omissions, and clarified food/beverage items with participants if required.

#### Food frequency questionnaire (DQESV2)

The DQESV2 was self-administered two weeks after completing the last one-day WFR to minimise the effect of recalling intake on responses. The semi-quantitative DQESV2 is categorised into three parts: 1) ten questions about the quantity and type of commonly consumed items which are used to provide additional detail for some of 74 food frequency items; 2) four questions based on a series of portion size photos for different food types used to scale intake data; 3) a list of 74 food/beverage items categorised under “cereal foods, sweets and snacks” (21 items), “dairy products, meat and fish” (15 items), “fruit” (13 items) and “vegetables (including fresh, frozen and tinned)” (25 items) with ten frequency responses ranging from “never” to “3 or more times per day”; followed by three additional questions to quantify intake of alcoholic beverages. Further detail on the DQESV2 instrument is provided by Hodge et al. [5]. The DQESV2 was designed to assess intake over the preceding 12 months, however in this study participants were instructed to complete the questionnaire based on their intake in the preceding one month for the purpose of using the questionnaire to measure recent dietary intake during an intervention trial. For example, the food frequency section of the DQESV2 asked participants *‘Over the last 1 month, on average, how often did you eat the following foods?’* Participants were not provided with a specific definition of the number of days in a month. Respondents were also invited to complete a second DQESV2 one week later, to study the reproducibility of the questionnaire. A dietitian collected and checked all questionnaires for errors and missing responses at the time of administration.

#### Nutrient analysis

Completed DQESV2 forms were sent to Cancer Council Victoria for analyses using specialist software based on the Australian NUTTAB 1995 food composition database, which yielded total daily intakes for the selected nutrients (*total energy, protein, total fat, saturated fat, carbohydrate, sugars, dietary fibre, and alcohol*). To ensure comparability, participants’ WFR were also analysed using the NUTTAB 1995 database in the nutrient analysis software Foodworks Professional Edition version 6 (Xyris Software, Highgate Hill, Queensland, Australia), from which, total daily intakes for the selected nutrients were obtained through an average of the five days of records (WFR Mean).

#### Fruit and vegetable servings

The gram weight of all fruits, vegetables and fruit or vegetable juices reported in the DQESV2 and WFR were summed independently. Totals from the WFR were then divided by five to yield the average daily intake in grams from the five days of records (WFR Mean). The number of daily fruit servings and vegetable servings were then calculated separately for both the DQESV2 and WFR Mean, by dividing whole fruit by 150 g, whole vegetables by 75 g, and fruit or vegetables juices by 130.9 grams (i.e. 125 mL * 1.046 specific gravity) [17], [18].

### Statistical Analysis

All statistical analyses were performed using SPSS (version 19 for Windows, IBM Corporation, Chicago, Illinois, United States), except weighted kappa statistics which were computed using SAS version 9.2 (SAS Institute, North Carolina, United States). The basal metabolic rate (BMR) of study participants was calculated using Schofield equations based on body weight, age and gender [19]. Under- and over-reporters were then identified as the top and bottom 2.5% of the distribution for the ratio of reported energy intake to BMR (EI_rep_:BMR). Statistical methods commonly used in validation studies of FFQs include correlation as well as measures of agreement such as weighted kappa on quintiles of intake and the Bland and Altman methods [1], [2]. Data on participants’ intake of nutrients and fruit and vegetable servings from the WFR Mean and DQESV2, were checked for normality. Pearson correlation coefficients (or Spearman rank coefficients for non-normal data) were then computed to measure the direction and strength of the linear relationship between methods for both the crude nutrient and fruit and vegetable intakes as well as those adjusted for energy using the residual method described by Willet et al. [20]. Correlation coefficients however, only describe the relationship between methods, and due to between subject variations in intake, related to variables such as age and gender, usually yield significant positive coefficients. To address this, the agreement between methods was calculated as the mean and standard deviation of the difference between methods. Using the methods proposed by Bland and Altman, the difference between methods (WFR Mean – DQESV2) was then plotted against the average ([WFR Mean+DQESV2]/2), for each nutrient and fruit and vegetable servings to permit judgement of the degree of variability in the difference between methods at varying levels of intake [21]. All variables for the difference between the methods were checked for normality. The 95% limits of agreement (LOA) were then calculated as the mean difference ± t (n-1, 0.025)*SD of the difference to assess the acceptability of agreement which is based on clinical judgement of the LOA rather than set thresholds or cut-points [21]. The regression equation *Difference = *a+β*(Average)* was calculated for each nutrient and fruit and vegetable servings, with the beta-coefficient (β) value for the slope used to indicate the degree of over- or under-estimation (agreement or difference) over the level of intake (average). Quintiles of intake from each method (WFR Mean and DQESV2) were calculated for each nutrient and fruit and vegetable servings, with the agreement between these quintile classifications compared using the weighted kappa statistic. To test the reproducibility of the DQESV2, nutrient and fruit and vegetable servings from the first and second DQESV2 administrations were compared using intra-class correlations (ICC) and the weighted kappa statistic for quintile classifications, including 95% confidence intervals, to provide measures of both consistency and agreement between repeat administrations [2].

## Results

### Validity

From a total of 109 participants recruited, 102 (93.6%) completed the study and were included in the analyses. Of these, sixty six participants were female (64.7%) and thirty six were male (35.3%), the mean (SD) age was 23.5 (4.1) years (range 18–34 years) and BMI 23.0 (3.3) kg/m^2^ (range 16–37 kg/m^2^). Of the seven excluded participants, three did not follow the study protocol, two lost contact and two withdrew. Cut-offs for the top and bottom 2.5% of the distribution for the EI_rep_:BMR ratio were >2.10 and <0.68 for the five-day average of the WFR (WFR Mean), compared with >2.15 and <0.53 for the DQESV2. A total of seven participants were outside these cut-offs, ranging from 0.56 to 2.42 in the WFR Mean and 0.41 to 2.25 for the DQESV2. As the exclusion of these participants from the analysis did not influence the findings, all participants were included in the analyses presented. Due to observed differences in validity and reproducibility findings between genders, the following results are presented separately for males and females.

Table 1 shows the mean and standard deviation for nutrient intakes and fruit and vegetable servings from the five-day average of the WFR (WFR Mean) and the DQESV2 for males and females, with correlation coefficients for crude and energy adjusted intakes. Estimates for nutrient intakes from the WFRs were all higher than those estimated from the DQESV2 among females, except for alcohol, while most estimates were lower among males. Among both genders, estimates from the WFRs for fruit servings and fruit servings including fruit juice were all lower than the DQESV2, while vegetable servings were all higher (Males 3.1 vs. 1.5; Females 3.8 vs 1.8). All nutrients and fruit and vegetable servings showed significant correlations between the two dietary assessment methods (*P*<0.05), except protein intake in males.

**Table 1:** Consistency between the five-day average of the weighed food records (WFR Mean) and the food frequency questionnaire (DQESV2), among males (n = 36) and females (n = 66).

Nutrient	Gender	WFR Mean x¯ (sd)	DQESV2 x¯ (sd)	*r*	*r* (Energy adjusted)
*Energy (kJ)*	Males	9313.3 (1793.8)	9414.7 (3111.5)	0.40*	–
	Females	7367.0 (2070.5)	6467.4 (2264.0)	0.47***	–
*Protein (g)*	Males	105.1 (25.6)	109.8 (39.8)	0.21	0.20
	Females	77.0 (22.9)	74.9 (31.3)	0.56***	0.56***
*Total fat (g)*	Males	85.4 (23.7)	91.4 (37.0)	0.35*	0.47**
	Females	69.2 (28.1)	59.0 (25.2)	0.52***	0.49***
*Saturated fat (g)*	Males	35.1 (12.4)	38.5 (16.7)	0.58***	0.64***
	Females	25.5 (10.4)	22.3 (10.9)	0.55***	0.50***
*Carbohydrate (g)*	Males	237.5 (56.8)	221.6 (74.0)	0.52**	0.40*
	Females	200.0 (61.4)	167.7 (62.9)	0.42***	0.64***
*Sugars (g)*	Males	72.9 (25.1)	86.3 (28.5)	0.59***	0.48**
	Females	80.4 (32.8)	73.5 (29.5)	0.50***	0.68***
*Dietary fibre (g)*	Males	23.1 (6.0)	22.3 (8.6)	0.38*	0.47**
	Females	24.4 (9.2)	19.6 (6.2)	0.49***	0.73***
*Alcohol (g)*	Males	13.3 (31.7)	15.6 (22.9)	0.76*** †	0.78*** †
	Females	5.4 (11.9)	7.0 (10.9)	0.70*** †	0.71*** †
**Food group**					
*Fruit servings*	Males	0.7 (0.6)	1.2 (0.9)	0.59***	0.40*
	Females	1.5 (1.1)	1.6 (1.0)	0.58***	0.69***
*Fruit servings (including fruit juice)*	Males	1.5 (1.2)	1.9 (1.0)	0.62***	0.42*
	Females	1.9 (1.2)	2.0 (1.1)	0.56***	0.68***
*Vegetables servings*	Males	3.1 (1.6)	1.5 (0.8)	0.45**	0.38*
	Females	3.8 (1.9)	1.8 (0.9)	0.53***	0.61***

* *P*<0.05.

** *P*<0.01.

*** *P*<0.001.

† Spearman’s Rho calculated, as data from both the DQESV2 and WFR Mean did not follow the normal distribution.

Table 2 presents results on the agreement between methods including the mean difference with 95% LOA, difference as a percentage of the WFR Mean, and coefficients for regression of the difference on the average. The mean difference was less than 20% of the WFR Mean for all nutrients, except alcohol intake in females. However, the 95% LOA indicated that at the individual level, the differences between the methods were quite large. For example, the DQESV2 under-estimated energy intake at the group level by 1% in males and 12% in females, however the DQESV2 could under-estimate energy intake by as much as 5806 kJ in males or 5370 kJ in females, and over-estimate energy intake by 6009 kJ in males or 3571 kJ in females, among 95% of the study population (Table 2). For fruit and vegetable servings, the mean differences were quite large, such that, at the group level, the DQESV2 over-estimated fruit servings by 82% in males and 9% in females, or by 28% and 4% respectively when fruit juice was included, while the DQESV2 under-estimated vegetable servings by 52% and 53% among males and females, respectively. Further, at the individual level, i.e. among 95% of the population, the DQESV2 could under-estimate vegetable servings by as much as 4.6 servings in males or 5.2 servings in females (Table 2).

**Table 2:** Agreement between the five-day average of the weighed food records (WFR Mean) and the food frequency questionnaire (DQESV2), among males (n = 36) and females (n = 66).

Nutrient	Gender	Difference *WFR Mean – DQESV2* † x¯ (sd)	95% LOA‡	% difference§	β
*Energy (kJ)*	Males	−101.4 (2912.8)	−6008.5–5805.8	−1.1	−0.75**
	Females	899.6 (2238.6)	−3570.9–5370.0	12.2	−0.12
*Protein (g)*	Males	−4.6 (42.4)	−90.7–81.4	−4.4	−0.70*
	Females	2.1 (26.4)	−50.6–54.8	2.7	−0.39**
*Total fat (g)*	Males	−6.0 (36.2)	−79.5–67.5	−7.0	−0.64**
	Females	10.1 (26.3)	−42.4–62.7	14.7	0.14
*Saturated fat (g)*	Males	−3.4 (13.9)	−31.5–24.7	−9.7	−0.37*
	Females	3.1 (10.1)	−16.9–23.2	12.3	−0.07
*Carbohydrate (g)*	Males	15.9 (65.5)	−116.9–148.7	6.7	−0.34
	Females	32.2 (66.9)	−101.3–165.7	16.1	−0.04
*Sugars (g)*	Males	−13.4 (24.5)	−63.1–36.4	−18.3	−0.16
	Females	6.8 (31.1)	−55.3–69.0	8.5	0.14
*Dietary fibre (g)*	Males	0.8 (8.5)	−16.4–17.9	3.3	−0.50*
	Females	4.8 (8.2)	−11.6–21.2	19.6	0.52***
*Alcohol (g)*	Males	−2.3 (23.9)	−50.7–46.1	−17.2	−0.58∥
	Females	−1.6 (9.4)	−20.3–17.2	−28.9	−0.14∥
**Food group**					
*Fruit servings*	Males	−0.6 (0.7)	−2.0–0.9	−82.1	−0.52**
	Females	−0.1 (1.0)	−2.1–1.8	−8.9	0.13
*Fruit servings (including fruit juice)*	Males	−0.4 (1.0)	−2.4–1.6	−27.9	0.30
	Females	−0.1 (1.1)	−2.3–2.1	−3.8	0.10
*Vegetables servings*	Males	1.6 (1.5)	−1.4–4.6	52.1	0.89***
	Females	2.0 (1.6)	−1.2–5.2	52.7	0.88***

* *P*<0.05.

** *P*<0.01.

*** *P*<0.001.

† All variables for the difference between methods followed the normal distribution.

‡ Mean difference ± t (n-1, 0.025)*SD difference.

§ [(WFR Mean – DQESV2)/WFR Mean]*100%.

∥ Due to significant heteroscedasticity, co-efficient was calculated on log transformed data for positively skewed distributions with zero values, LG10*(Difference+60) = a+b(*LG10*(Average+60)*.

**Figure 1:**
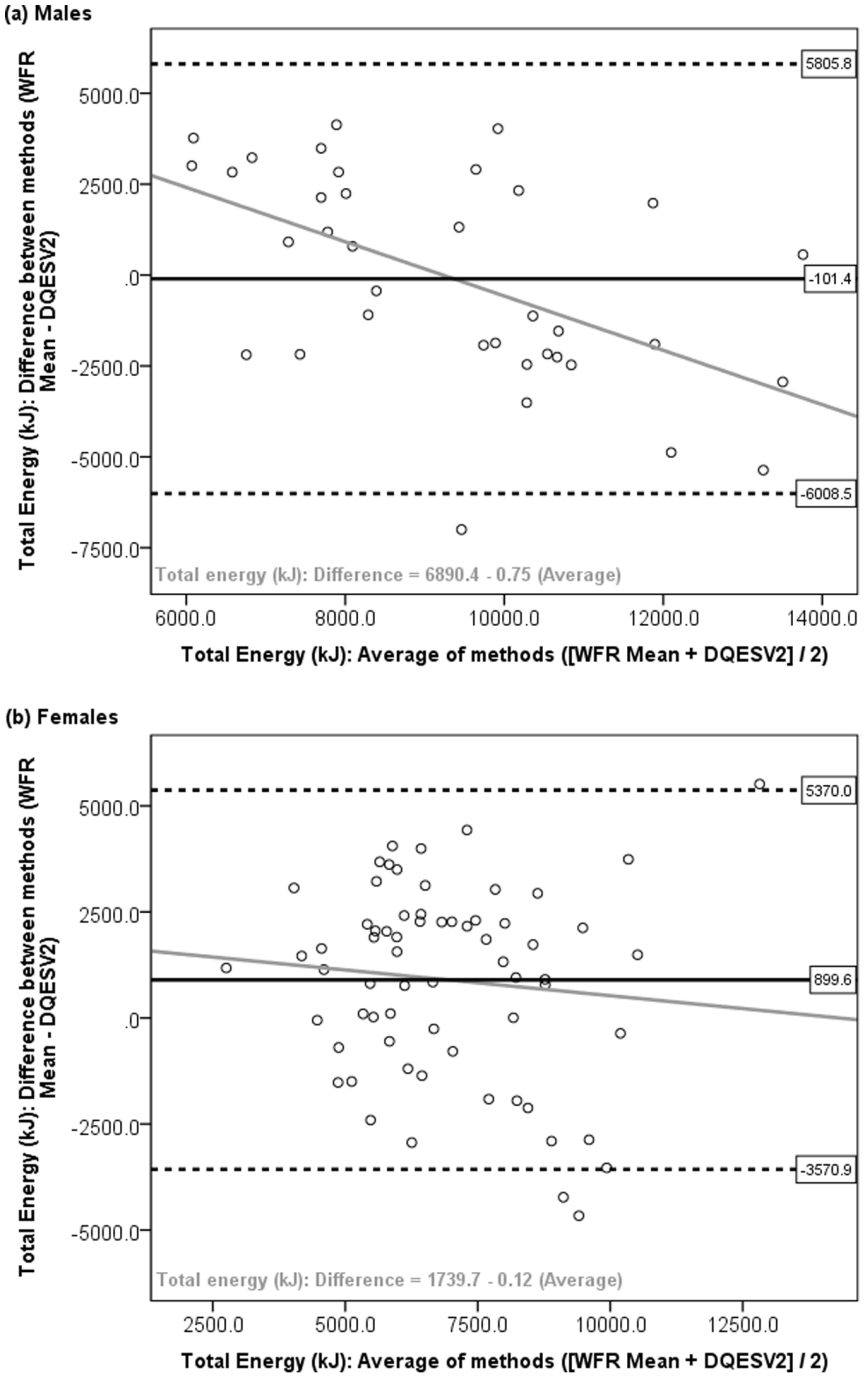
Bland and Altman plot: Difference in total energy intake in kilojoules (kJ) from the DQESV2 and WFR Mean, against the average of the two methods, among (a) males and (b) females. Horizontal lines represent the mean difference (solid black) and 95% limits of agreement (dotted lines). Grey line represents the regression line for the equation *Difference = a+β(Average)*.

**Figure 2:**
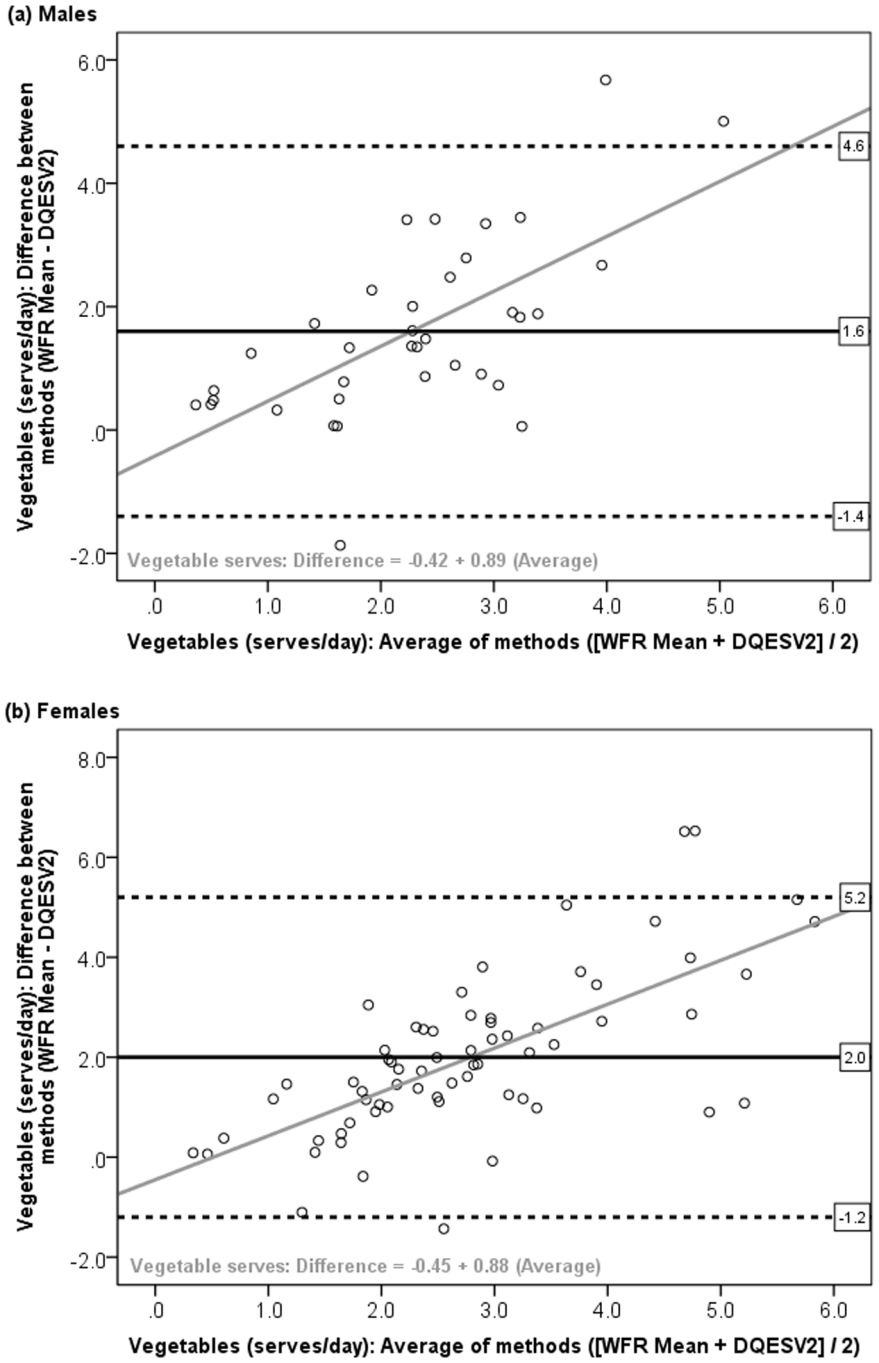
Bland and Altman plot: Difference in vegetables (servings per day) from the DQESV2 and WFR Mean, against the average of the two methods, among (a) males and (b) females. Horizontal lines represent the mean difference (solid black) and 95% limits of agreement (dotted lines). Grey line represents the regression line for the equation *Difference = a+β(Average)*.

Through visual inspection of the Bland and Altman plots (i.e. the difference between methods (WFR Mean – DQESV2) plotted against the average ([WFR Mean+DQESV2]/2), a tendency toward poorer agreement in nutrient intakes between methods was observed with higher levels of intake, i.e. the difference between the DQESV2 and WFR Mean estimates became larger as nutrient intake increased. Figures 1 and 2 present the Bland and Altman plots for total energy and vegetable servings in males and females, with the regression line equation *Difference = a+β(Average)*. Larger beta coefficients indicated poorer agreement with increasing level of intake (Table 2). Most coefficients were negative, indicating that the DQESV2 provided a greater over-estimation of intake at higher intakes, as found for total energy in males (β = −0.75; Figure 1a) and protein (Males β = −0.70; Females β = −0.39). However for vegetable servings, the DQESV2 provided a greater under-estimation of intake at higher intakes, indicated by the significant positive coefficients (Males β = 0.89; Females β = 0.88, *P*<0.001), as shown in Table 2 and illustrated in Figures 2a and 2b.

Agreement within DQESV2 quintile classifications in the nutrients and fruit and vegetable servings is presented in Table 3. For all nutrients and fruit and vegetable servings, gross misclassification by four quintiles did not occur in more than 3% of the sample, except for protein intake in males, and over 60% were within one quintile classification, except for total fat and dietary fibre intakes in males (55% and 56%, respectively). Weighted kappa agreement ranged between 0.18 for protein and dietary fibre intake in males (slight agreement) and 0.54 for fruit servings in males (moderate agreement [22]).

**Table 3:** Agreement between quintiles of intake from WFR Mean and DQESV2, among males (n = 36) and females (n = 66).

Nutrient	Gender	Percent (%) allocation by quintile					Weighted Kappa	95% CI
		Exact	Adjacent*	+/−2 Quintiles	+/−3 Quintiles	GM†		
*Energy (kJ)*	Males	31	42	19	6	3	0.32	0.11–0.52
	Females	26	36	29	9	0	0.24	0.08–0.40
*Protein (g)*	Males	25	36	28	6	6	0.18	−0.05–0.40
	Females	50	26	15	8	1	0.47	0.30–0.63
*Total fat (g)*	Males	36	19	31	11	3	0.21	−0.02–0.45
	Females	35	29	30	5	1	0.31	0.15–0.47
*Saturated fat (g)*	Males	39	41	14	3	3	0.43	0.22–0.65
	Females	26	44	21	8	1	0.27	0.12–0.43
*Carbohydrate (g)*	Males	20	47	22	11	0	0.21	0.00–0.42
	Females	24	40	27	9	0	0.24	0.08–0.39
*Sugars (g)*	Males	36	44	14	6	0	0.43	0.24–0.63
	Females	30	44	18	8	0	0.35	0.21–0.49
*Dietary fibre (g)*	Males	25	31	36	6	3	0.18	−0.06–0.41
	Females	21	47	27	5	0	0.27	0.13–0.42
*Alcohol (g)*	Males	30	53	17	0	0	0.42	0.25–0.59
	Females	38	53	5	5	0	0.50	0.39–0.62
**Food group**								
*Fruits servings*	Males	47	33	20	0	0	0.54	0.36–0.72
	Females	36	41	20	2	2	0.43	0.28–0.57
*Fruit servings* *(including fruit juice)*	Males	39	33	25	3	0	0.4211	0.21–0.63
	Females	36	44	15	5	0	0.4465	0.30–0.59
*Vegetables servings*	Males	33	42	11	11	3	0.3158	0.08–0.55
	Females	36	36	17	9	2	0.3511	0.19–0.51

* Disagreement by one quintile.

† Gross misclassification, i.e. disagreement by four quintiles.

### Reproducibility

A total of 77 participants completed a second DQESV2. These participants were not statistically different in age, gender or BMI to those who did not complete a second DQESV2. Table 4 presents the mean daily intakes of all nutrients and fruit and vegetable servings, with corresponding ICC coefficients and weighted kappa coefficients with 95% confidence intervals. All ICC coefficients showed significant positive correlations between the first and second DQESV2 administrations (*P*<0.01), ranging from 0.54 for fruit servings (including fruit juice) in males to 0.91 for protein intake in females. Weighted kappa coefficients ranged from 0.43 (moderate agreement) for dietary fibre in males to 0.74 (substantial agreement) for protein intake in females.

**Table 4:** Reproducibility of the DQESV2 among male (n = 27) and female (n = 50) participants completing a second DQESV2.

Nutrient	Gender	First DQESV2 x¯ (sd)	Second DQESV2 x¯ (sd)	ICC†	Weighted Kappa	95% CI
*Energy (kJ)*	Males	9145.2 (3360.7)	8404.8 (3020.1)	0.77***	0.62	0.44–0.79
	Females	6633.2 (2288.5)	6332.7 (2603.6)	0.86***	0.68	0.54–0.81
*Protein (g)*	Males	106.2 (37.7)	99.1 (36.9)	0.78***	0.64	0.47–0.82
	Females	75.6 (32.2)	72.3 (34.0)	0.91***	0.74	0.62–0.85
*Total fat (g)*	Males	88.1 (39.4)	79.5 (32.3)	0.78***	0.68	0.52–0.84
	Females	61.0 (25.9)	58.3 (27.1)	0.86***	0.69	0.55–0.83
*Saturated fat (g)*	Males	36.8 (17.9)	32.8 (14.9)	0.83***	0.68	0.52–0.84
	Females	23.3 (11.4)	22.2 (11.4)	0.90***	0.72	0.60–0.84
*Carbohydrate (g)*	Males	221.6 (80.0)	206.9 (73.2)	0.79***	0.61	0.42–0.81
	Females	174.2 (64.3)	166.7 (76.4)	0.86***	0.68	0.53–0.82
*Sugars (g)*	Males	84.0 (30.7)	80.0 (30.0)	0.66***	0.56	0.35–0.78
	Females	75.8 (31.8)	71.3 (28.0)	0.83***	0.52	0.36–0.68
*Dietary fibre (g)*	Males	21.6 (7.5)	20.2 (7.0)	0.81***	0.43	0.20–0.66
	Females	20.2 (6.1)	19.6 (8.3)	0.74***	0.46	0.29–0.63
*Alcohol (g)*	Males	12.6 (17.8)	10.7 (11.9)	0.79*** ‡	0.65	0.48–0.83
	Females	6.1 (8.7)	5.5 (8.1)	0.88*** ‡	0.73	0.60–0.85
**Food group**						
*Fruit servings*	Males	1.1 (0.7)	1.2 (0.8)	0.72***	0.63	0.41–0.86
	Females	1.6 (1.0)	1.7 (1.1)	0.86***	0.71	0.60–0.82
*Fruit servings* *(including fruit juice)*	Males	1.7 (0.9)	1.9 (1.2)	0.54**	0.51	0.28–0.74
	Females	2.1 (1.2)	1.9 (1.2)	0.72***	0.53	0.38–0.68
*Vegetable servings*	Males	1.4 (0.8)	1.6 (1.0)	0.71***	0.58	0.37–0.80
	Females	1.8 (0.9)	1.8 (0.9)	0.69***	0.53	0.36–0.70

** *P*<0.01.

*** *P*<0.001.

† ICC calculation based on single measures of absolute agreement from the two-way mixed model.

‡ ICC calculated using the square root of alcohol from the first and second DQESV2 as data did not follow the normal distribution.

## Discussion

This study aimed to examine the relative validity and reproducibility of a semi-quantitative food frequency questionnaire (DQESV2) against five-days weighed food records, over a one month period in young adults. Comparing the two dietary assessment methods, all nutrients, fruit servings and vegetable servings were found to be significantly correlated (*P*<0.05), except protein intake in males. Correlation coefficients reported in this study were substantially higher than those found in an earlier study assessing the validity of an Australian FFQ against food records in 9–16 year old children over a six month period [23]. The limits of agreement indicated that at an individual level the differences in estimates produced by the two methods could be very large. Hence in terms of validity, the DQESV2 did not provide an accurate estimate for a single individual’s intake, although at the group level, the DQESV2 estimated intake within +/−20% for all nutrients studied, except alcohol intake in females, in this population. Previous validation studies for the DQESV2 have also reported total energy and macro-nutrients (protein, fat and carbohydrate) to vary by up to 20% between methods [5], [6]. Reported alcohol intake in the DQESV2 was higher than that reported in the WFR for both males and females. This appeared to be due to a lower number of participants reporting no alcohol intake in their DQESV2, compared with their WFR (22% vs. 51%), which may have been due to the specific days selected for completing the WFR omitting days when alcohol was consumed or that DQESV2 responses were based on a ‘usual’ month rather than the last month specifically.

Food groups have not previously been reported in validation studies of the DQESV2. Increasingly it is being recognised that taking a reductionist nutrient-centric approach to study diet-disease relationships is less useful than a food-based approach. The findings of the present study were similar to another Australian study that used a different FFQ (adapted from that used in the Nurses’ Health Study) in 96 subjects who completed 6 two-day weighed food records over a one-year period, where fruit and vegetable intake was shown to be correctly classified to within one quartile for 79% and 69% of subjects, respectively [24]. However the FFQ used in that study over-estimated vegetable intake, while the DQESV2 underestimated intake [24]. Vegetable servings could be underestimated by as much as 5.2 servings in females or 4.6 servings in males, among 95% of the sample or by almost two servings at the group level, in this population. Given Australian national dietary recommendations are to consume at least five servings of vegetables per day [25], this means a substantial proportion of subjects meeting these recommendations would not be classified as such, and hence the DQESV2 should not be used to compare vegetable intake to national recommendations, among individuals or groups, in this population. The under-estimation of vegetable servings from the DQESV2 may partly be due to the scaling method used which is based on the short question *“How many different vegetables do you usually eat in a day?”* The median response to this question was 2–3 different vegetables per day, while the total daily equivalents from the frequency section of the DQESV2 indicated a mean 4.9 serves of vegetables per day, and hence for most subjects, vegetable intake would have been scaled down to the number of different vegetables they reported consuming daily. There were also a number of vegetables reported in the WFR that are not assessed by the DQESV2 including artichoke, asparagus, eggplant, celeriac, parsnip, radish, squash and corn, the most frequently consumed being corn (n = 17; 118 g/person) and eggplant (n = 10; 74 g/person), which if included may have improved estimates from the DQESV2. The DQESV2 slightly over-estimated fruit intake at the group level in males by around half a serving per day, although this over-estimation was attenuated when fruit juice was included. It is unlikely that this over-estimation was due to the scaling question *“How many pieces of fresh fruit do you usually eat per day?”*, as the median response to this question, 1–2 pieces per day, was consistent with the mean 1.9 total daily equivalent serves of fruit (excluding fruit juice) reported in the frequency section of the DQESV2. Worth noting however was that, similar to vegetables, there were also a number of fruits that were reported as consumed in the WFRs that were not included in the DQESV2, such as blueberries, cranberries, raspberries, cherries, grapes, guava, kiwi fruit, lychees, passion fruit, plum, prune, quince, and dried fruits. Of these, the most frequently consumed were kiwi fruit (n = 21; 152 g/person), grapes (n = 10; 215 g/person) and blueberries (n = 10; 128 g/person), which if included, may have increased the degree to which the DQESV2 over-estimated fruit intake.

Reproducibility of the DQESV2 has not been previously reported. This study found all reproducibility correlation coefficients to be significant (*P*<0.01), ranging from 0.54 for fruit servings (including fruit juice) in males to 0.91 for protein intake in females. These coefficients are substantially higher than those found from repeat administration of an FFQ one year apart on a sample of Australian women 22 to 79 years of age [26], or five months apart on a sample of 9–16 year olds [23]. This is unsurprising given that repeat administrations of the questionnaire were only one week apart and hence responses to the second questionnaire may have been biased by recall of responses to the first. Others have also reported reproducibility coefficients to be relatively high when questionnaires are repeated one month or less apart [2]. Consistency in reported protein, fat and carbohydrate intakes were found to be lower among males than females which was also demonstrated in an earlier reproducibility study of a similar FFQ administered to college students [27]. Further, agreement between repeat administrations, as assessed by Cohen’s weighted kappa statistic, was substantially higher than that found for a similar Australian FFQ administered to 9–16 year olds [23]. While sensitivity to change was not tested, the DQESV2 has good reproducibility for the foods and nutrients of interest in this population, indicating changes resulting from participation in an intervention research trial would be likely to be detected at the group level.

Weighed food records were used as the ‘gold standard’ method for comparison in this study, although this method is also subject to reporting bias. Human measurement error may also have occurred in the WFR when the young adults were asked to estimate their intake while eating out, although this was necessary for practical reasons and all estimated intakes were consistently transformed into gram weights using the current Australian food measures database [16]. The five one-day WFR were administered over one month, followed by a lapse of two weeks before administration of the DQESV2. While this meant the first one-day WFR was not within the one month period assessed by the first administration of the DQESV2, four one-day WFR were, and this permitted a ‘wash out’ period to minimise the effect of recalling intake on responses to the DQESV2. Similarly, a one week ‘wash out’ period was used for the second administration of the DQESV2, and while this omitted the first week of the one month reference period, this was necessary to minimise the effect of recalling responses to the first DQESV2 on responses to the second. Dietary records were completed by participants between mid August and the end of October 2011, and thus excluded important holidays and study vacation periods which may have affected usual intake. A slightly higher proportion of WFRs were from weekdays (66%) compared with weekend days (34%), although it is likely that among young adults, Fridays (upon which 20% of WFRs were conducted) may more closely reflect weekend dietary patterns rather than weekday patterns. Dietary intake estimates from the DQESV2 are limited by the use of a 1995 Australian food database, which has since been updated, the most recent being NUTTAB 2010 [18], although changes are largely in the number of products analysed and in some micronutrients, as a result of folate fortification for example, rather than macronutrient data used in the present study.

As the DQESV2 was not developed for a young adult population, certain foods consumed by the study participants were not represented in the DQESV2. These included sweets/candy, soft drinks, milk alternatives (i.e. almond, oat, rice), cream, oils, and some bakery items (i.e. doughnuts, English muffins, crumpets). The DQESV2 asks about butter and margarine spreads but not oils, and this may be why total fat intake among females was underestimated in the DQESV2 while estimates for saturated fats were more comparable between methods. As the DQESV2 only asks about slices of bread, participants may not have included other types of breads consumed (e.g. flat breads or bagels). Further, as the DQESV2 asks about specific brands of breakfast cereals, participants might not have included other cereal brands, despite them having similar nutritional content. These factors might explain why energy, carbohydrate and dietary fibre intakes were under-estimated by the DQESV2. It is possible the participants had a self interest in dieting for weight management (being a young, mostly female population) which could have led to some of the young adults under-eating during the study, although this should have been captured in both dietary assessment methods. Participants were also tertiary education students from a largely urban area of New South Wales, Australia, and hence this limits the applicability of validity and reproducibility findings to young adults outside of this population. Finally, this study only assessed the specific nutrients energy, protein, total fat, saturated fat, carbohydrate, sugars, dietary fibre and alcohol as well as fruit and vegetable servings, which are the focus of our proposed intervention studies.

One of the strengths of the current study is that five one-day WFR were used for comparison which is the gold standard for measuring dietary intake [1], [14]. In addition, measuring equipment, recording booklets and instructions were provided and explained to participants to facilitate accurate recording. Completed WFR and DQESV2 were both checked by a Dietitian for clarification and completion. The study assessed for under-reporting but found exclusion of these participants did not significantly change the results. As the study sample were reimbursed for their participation, the dietary habits and awareness might be closer to the general population than if volunteers were recruited for the study. However, as we recruited from a University population the subjects may have been more proficient in completing the DQESV2, compared with less educated groups.

In summary, the DQESV2 is appropriate for use in this population group for estimating intake of the studied nutrients, except alcohol intake among females, and of fruit servings when fruit juice is included, at the group level over a one month period. While the agreement between methods was poorer for alcohol generally, it is not clear which was the better method. The DQESV2 cannot be recommended for estimating vegetable servings or for estimating fruit servings at the individual or group level, unless servings of fruit juice are included, due to the poor agreement found between methods. However, the DQESV2 demonstrated good reproducibility for all nutrients studied and for fruit and vegetable servings among both males and females from this population. The ability to use the easily-administered DQESV2 to assess diet in this population will be beneficial, particularly given the recent availability of an electronic version of this questionnaire.
